# Anticancer Activity of Postbiotic Mediators Derived from Lactobacillus Rhamnosus GG and Lactobacillus Reuteri on Acute Lymphoblastic Leukemia Cells

**DOI:** 10.31661/gmj.v12i.3096

**Published:** 2023-08-19

**Authors:** Morteza Banakar, Shahroo Etemad-Moghadam, Roza Haghgoo, Majid Mehran, Mohammad Hossein Yazdi, Hadiseh Mohamadpour, Mahdiyar Iravani Saadi, Mojgan Alaeddini

**Affiliations:** ^1^ Dental Research Center, Dentistry Research Institute, Tehran University of Medical Sciences, Tehran, Iran; ^2^ Department of Pediatric Dentistry, Faculty of Dentistry, Shahed University, Tehran, Iran; ^3^ Biotechnology Research Center, Tehran University of Medical Sciences, Tehran, Iran; ^4^ Hematology Research Center, Shiraz University of Medical Sciences, Shiraz, Iran

**Keywords:** Cancer, Acute Lymphoblastic Leukemia, Probiotic, Postbiotic, Anticancer

## Abstract

Background: Leukemia remains a global health challenge, requiring the exploration of alternative therapies with reduced side effects. Probiotics, particularly Lactobacillus species, have gained attention because of their potential anticancer properties. This study investigated the anticancer and cytotoxic effects of postbiotic mediators (PMs) derived from Lactobacillus rhamnosus GG (LGG) and Lactobacillus reuteri (LR) on acute lymphoblastic leukemia (ALL) cells and peripheral blood mononuclear cells (PBMCs). Materials and Methods: The PMs were prepared by culturing LGG and LR strains and isolating the supernatant. The MTT assay assessed cell viability on ALL Jurkat cells and PBMCs, and apoptosis analysis was conducted using flow cytometry. Quantitative real-time PCR was also performed to analyze BAX, BCL-2, BCLX, FAS, and p27 gene expression levels. Results: The results showed that PMs derived from LGG and LR significantly reduced cell viability in Jurkat cells (P0.05) but not PBMCs (P0.05). Apoptosis analysis revealed an increase in apoptotic cells after PMs treatment. Nevertheless, gene expression analysis revealed no statistically significant difference between the treated and untreated groups in BAX, BCL-2, BCLX, FAS, and p27 gene expression levels (P0.05). Conclusion: Findings suggest that specific PMs derived from LGG and LR possess anticancer properties against ALL cells. This research highlighted the promise of PMs as a cutting-edge and less toxic adjuvant therapeutic strategy in cancer treatment.

## Introduction

Cancer continues to pose a substantial health burden worldwide, as evidenced by the approximately 19.3 million new cases and 10 million fatalities in 2020 [1]. Acute lymphoblastic leukemia (ALL) is the predominant form of childhood cancer, constituting approximately 25% of all malignancies diagnosed in the pediatric population [2]. Despite advances in understanding cancer biology and developing novel therapeutic strategies, the effectiveness of cancer treatment is still hindered by the toxicity and enduring adverse effects of traditional chemotherapy drugs, as well as the development of drug resistance [3]. As the prognosis of cancer has increased, the focus of treatment has shifted from rescuing patients at all costs to saving patients at the lowest cost [4].

Consequently, there is a growing interest in exploring alternative and complementary therapies that possess anticancer properties with reduced adverse effects on peripheral blood mononuclear cells (PBMCs).

Probiotics, living microorganisms providing health advantages to the host when given appropriately, have been extensively studied for their potential therapeutic applications in various diseases, including cancer [5]. Probiotics, particularly lactic acid bacteria (LAB) including Lactobacillus species, given their well-documented safety profile and beneficial effects on the human gut microbiome, have attracted considerable attention due to their beneficial effects on host health, including a potential role in preventing cancer and adjuvant cancer therapy [6][7]. Among these, Lactobacillus rhamnosus GG (LGG) and Lactobacillus reuteri (LR) have shown promise in reducing inflammation, modulating the immune system, and the growth of pathogenic bacterial [8][9].

The mechanisms of probiotic activity against cancer are multifactorial, involving immunomodulation, suppression of tumor cell proliferation, and the production of bioactive metabolites [10].

Recently, research has shifted focus from the use of live probiotic bacteria to the application of their metabolic byproducts, called postbiotics or postbiotic mediators (PMs). PMs are bioactive molecules including a heterogeneous group of compounds, consisting of proteins, peptides, cell wall components, and other metabolites, mediating the beneficial effects of probiotics on host physiology [11][12].

PMs offer several advantages over live probiotics, including enhanced stability, reduced risk of bacterial translocation, absence of antibiotic resistance concerns, reduced immune response, and lower risk of infection, especially in immunocompromised such as patients with ALL [12][13]. Recent evidence suggests that certain PMs derived from Lactobacillus species may possess anticancer properties [14]. For example, LGG-derived proteins and exopolysaccharides have antigenotoxic and cytotoxic potential to inhibit the proliferation and infiltration of colon cancer cells [15]. Similarly, LR-produced reuterin suppresses proliferation and induces cell death in various human cancerous cells [16][17]. However, the potential anticancer activity of PMs derived from LGG and LR against ALL remained largely unexplored. This study aimed to examine the anticancer and cytotoxic properties of PMs derived from LGG and LR on ALL cancer cells and PBMCs. We hypothesize that specific PMs may selectively target ALL cells without causing significant cytotoxicity to normal cells, thus representing a promising therapeutic approach for ALL treatment. By elucidating these PMs’ underlying molecular mechanisms of action, we hope to contribute to the increasing body of evidence supporting the potential application of PMs as effective, novel, and less toxic adjuvant therapeutic strategies in cancer therapy.

## Materials and Methods

Ethics Approval and Consent to Participate

The present study was carried out under the authorization and oversight of the National Institute for Medical Research Ethics Committee (IR.NIMAD.REC.1400.148) and the Ethics Committee of Tehran University of Medical Sciences (IR.TUMS.DENTISTRY.REC.1400.191). The procedures were conducted following the appropriate guidelines and regulations.

Preparation of Postbiotics

LGG (ATCC 53103) and LR (ATCC 23272) were purchased from the Pasteur Institute of Iran (Tehran, Iran) and maintained on MRS agar (Merck, Darmstadt, Germany). The Lactobacillus strains were grown in MRS broth (Merck, Darmstadt, Germany) for 48 hours at 37±1 °C in a CO2 incubator before each experiment. After incubation, the supernatant and cell pellet from Lactobacillus cultures were separated by centrifugation at 10,000×g (8,000 rpm) for 10 minutes at 4 °C. The supernatant was filtered through a 0.22 µm polyethersulfone membrane syringe filter (Millipore, Burlington, USA) and neutralized with 5 M sodium hydroxide to achieve a physiological pH of 7.2-7.4 [18][19].

Cell Culture and Maintenance

Immortalized ALL (ATCC TIB-152) cell line was prepared from the Pasteur Institute, Tehran. The cell line was maintained in a culture medium consisting of RPMI, L-glutamine, 10 mM HEPES, 23.8 mM sodium carbonate, and 10% fetal bovine serum (FBS). Incubation took place at 37°C, with an environment of 95% humidity and 5% CO2. The culture conditions were monitored and adjusted daily if necessary.

PBMCs were isolated from 5 mL of peripheral blood samples obtained from healthy donors. Written and informed consent was obtained from the volunteer. Buffy coats were diluted with phosphate-buffered saline (PBS) in the rate of 1:1, and 3ml of ficolEX (DNA biotech, Tehran, Iran) was added to the buffy coat for centrifuging at 400× g for 20 min. This stage consisted of four layers. The isolation of PBMCs involved subjecting the second layer to a triple wash using PBS at 100×g for a duration of 10 minutes at ambient temperature. The purified PBMCs were then cultured at 37°C with 5% CO2, utilizing RPMI-1640 medium (Sigma, St Louis, USA) enriched with 10% (v/v) heat-inactivated FBS and 100 U/ml penicillin-streptomycin.

MTT Assay for Cytotoxicity Evaluation

The Jurkat cell line was plated in 96-well microplates at a density of 25,000 cells/mL and incubated at 37°C with a 5% CO2 atmosphere for cytotoxicity assessment. Following 24 hours of incubation, a serial dilution of the percentage of a wide range from 100% (V/V) to 25% (V/V) post-biotic mediators (PM) produced by LGG and LR strains was added to the complete growth medium. Cells not treated with PM served as controls.

The cells were incubated for 24 and 48 intervals. At each instance, 20 μL of 5 mg/mL MTT solution (3-(4,5-dimethylthiazol-2-yl)-2,5-diphenyl tetrazolium bromide) (Sigma-Aldrich, Steinheim, Germany) in PBS was added to each well. The plates were incubated in the dark for a duration of 4 hours before centrifugation at 2000×g for 5 minutes to separate formazan crystals. Subsequently, 170 μL of growth medium was extracted from each well, and the resulting formazan crystals were dissolved in 100 μL of dimethyl sulfoxide (DMSO) (Fisher Scientific, UK) for 15-30 minutes. The absorbance of the formazan dye was measured by a plate reader (M491-Epoch reader, Bio Tek Instruments, Inc., Winooski, VT, USA) at 570 nm. The experiment was carried out in triplicate and replicated three times.

Cell viability was calculated as a percentage according to the following equation: [(A_sample - A_blank) / (A_control - A_blank)] × 100%, where A_sample represents the absorbance of cells treated with PMs, A_blank represents the absorbance of PMs, and A_control represents the absorbance of untreated cells. The half-maximal inhibitory concentration (IC50) was determined [19].

Apoptosis Analysis by Flow Cytometry

To investigate the induction of apoptosis by PMs derived from LGG and LR, ALL cells were treated with PMs at a concentration determined to be the IC50 value for 24 hours. After the incubation periods, the cells were harvested by centrifugation at 2500×g for 5 minutes and rinsed twice with cold PBS. The cell pellets were then resuspended in 100 μL of binding buffer (10 mM HEPES, pH 7.4, 140 mM NaCl, 2.5 mM CaCl2), and stained with 5 μL of Annexin V-FITC (fluorescein isothiocyanate) and 5 μL of propidium iodide (PI) solution (BD Biosciences, USA) for 15 minutes at room temperature while being kept in the dark. Each sample was supplemented with 400 μL of binding buffer, and the stained cells were subsequently analyzed using a flow cytometer (BD FACSCanto II, USA) within a time frame of one hour.

A minimum of 10,000 events were recorded for each sample, and the data were analysed using FlowJo software (BD Biosciences, USA). The apoptotic cells were characterized by their positive staining for Annexin V-FITC and negative staining for PI, indicating early apoptosis, or by their positive staining for both Annexin V-FITC and PI, indicating late apoptosis. The apoptotic cell percentage in each of the treatment groups was compared to that of the control group to assess the pro-apoptotic impact of the PMs on ALL cells. The experiments were conducted in triplicate and replicated thrice to ensure reproducibility.

RNA Isolation, cDNA Synthesis, and Quantitative Real-Time PCR Analysis

The extraction of total RNA from both treated and untreated Jurkat cells was performed using the RNeasy Mini Kit (GeneAll Biotechnology Co, Korea) according to the manufacturer’s instructions.

The concentration and purity of RNA were evaluated using a NanoDrop instrument (M491-Epoch reader, Bio Tek Instruments, Inc., Winooski, VT, USA). A Revert Aid First Strand cDNA Synthesis Kit (Thermo Fisher Scientific, US) was utilized to synthesize complementary DNA (cDNA) from 2μg of total RNA following the instructions provided by the manufacturer. The cDNA synthesis was conducted using a 20 μL volume comprising 1 μg of total RNA, 2 μL of 10x reverse transcription (RT) buffer, 0.8 μL of 25x deoxyribonucleotide triphosphate (dNTP) mix (100 mM), 2 μL of 10x RT random primers, 1 μL of MultiScribe Reverse Transcriptase (50 U/μL), 1 μL of RNase inhibitor (20 U/μL), and nuclease-free water.

The reverse transcription reaction was conducted using a thermal cycler under the specified conditions: incubation at 25°C for a duration of 10 minutes, followed by incubation at 37°C for 60 minutes, and finally, incubation at 85°C for 5 minutes. The experimental procedure involved the utilization of the PowerUp SYBR Green Master Mix (Takara, Kyoto, Japan) Detection System and Software (Real-time PCR Roche Light Sycler 96) to perform quantitative reverse transcription polymerase chain reaction (qRT-PCR) analysis.

The reaction mixture for qRT-PCR consisted of 20 μL in total.

This included 10 μL of PowerUp SYBR Green Master Mix, 1 μL of cDNA template, 1 μL of each forward and reverse primer (at a concentration of 10 μM), and 7 μL of nuclease-free water. The thermal cycling protocol consisted of the following steps: an initial activation of uracil-DNA glycosylase (UDG) at a temperature of 50°C for a duration of two minutes, followed by activation of the dual-lock DNA polymerase at 95°C. This was then followed by 40 cycles of denaturation at 95°C for 15 seconds and annealing/extension at 60°C for 1 minute. The primer sequences for the target genes (BAX, BCL-2, BCLX, FAS, and p27) and the reference gene (ACTB) were designed utilizing the Primer-BLAST tool provided by the National Center for Biotechnology Information (NCBI).

The analysis of the relative expression levels of target genes was conducted according to the method proposed by Livak and Schmittgen [20]. The experiments were conducted three times, and the data were reported as the mean ± standard deviation (SD).

Statistical Analysis

The experiments were conducted in triplicate, each experiment was performed independently. The resulting data were reported as the mean value along with the standard deviation (SD). The statistical method of one-way analysis of variance (ANOVA) was utilized, followed by the application of Tukey’s post hoc test, to identify significant differences in the analyses. Analysis of flow cytometry data was done by t-test. The gene expression level was also determined as n-fold changes relative to the calibrator. The statistical significance of the results was demonstrated by a P-value less than 0.05.

## Results

PMs Correlated with Cell Viability

**Figure-1:**
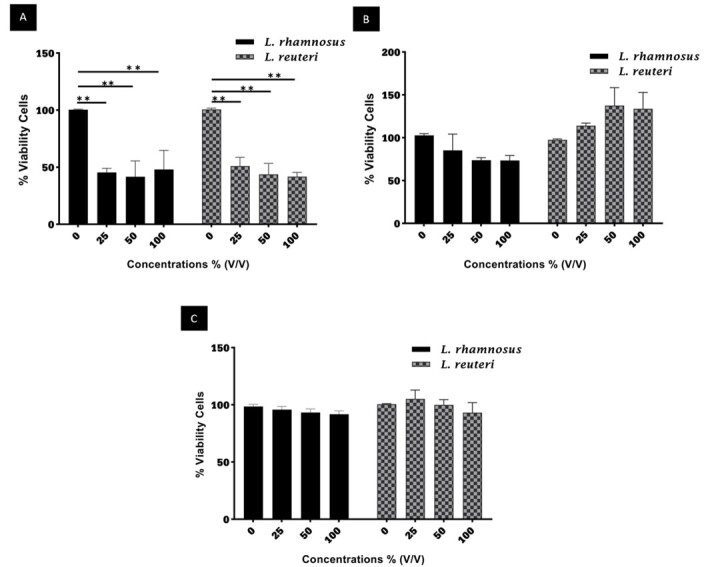
Figure-1 A-C. PMs Correlated with Cell Viability. A: The impact of PMs on Jurkat cells after 24h. B: The impact of PMs on Jurkat cells after 48h. C: The impact of PMs on peripheral blood monocular cells after 24h. (*P<0.05; **P<0.01).

The effect of PMs derived from LR and LGG on acute lymphoblastic leukemia (Jurkat) cells and PBMCs was evaluated using the MTT assay. Cells were subjected to different concentrations of the PMs (100%, 50%, and 25% (v/v)) for 24 hours and 48 hours (Figure-1).

Effects of LGG and LR PMs on Jurkat Cell Viability

The viability of Jurkat cells, when exposed to PMs of LGG at concentrations of 100%, 50%, and 25% (v/v), exhibited a significant decrease after 24 hours in comparison to the control group (P<0.05), with mean viabilities of 48.09%, 41.70%, and 45.39%, respectively. Similarly, LR PMs at 100%, 50%, and 25% (v/v) also significantly reduced the viability of Jurkat cells compared to the control group (P<0.05), with mean viabilities of 41.82%, 43.82%, and 51.00%, respectively. After a duration of 48 hours, the viability of Jurkat cells, when subjected to LGG and LR postbiotic mediators at various concentrations, did not show any significant difference among the concentrations investigated (P>0.05).

Effects of LGG and LR PMs on Normal Cell Viability

The viability of PBMCs was assessed through MTT assays following a 24-hour exposure to LGG and LR PMs. Following 24-hours, there was not any significant difference in cellular viability between the experimental groups subjected to LGG PMs and the control group (P>0.05). The findings of this study demonstrate that the PMs derived from LGG and LR did not exhibit a statistically significant cytotoxic impact on PBMCs at the concentrations examined.

Gene Expression

**Figure-2:**
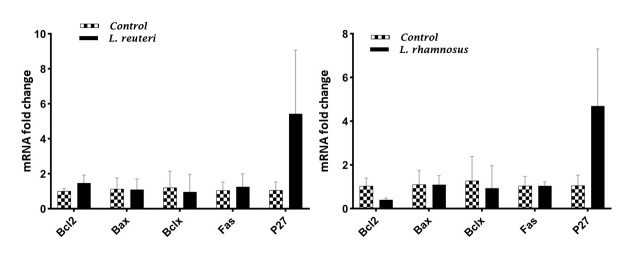
Figure-2. Expression of the genes in treated with PMs and untreated Jurkat cells

The gene expression levels of BAX, BCL-2, BCLX, FAS, and p27 in Jurkat cells untreated and treated with PMs from IC50 (100v/v) LGG and IC50 (25v/v) LR after 24 hours were assessed by qRT-PCR (Figure-2). p27 expression increased notably in both groups treated with PMs. Nevertheless, there was no significant difference in BAX, BCL-2, BCLX, FAS, and p27 gene expression levels between the treated and untreated groups for either LR or LGG (P>0.05).

Flow Cytometry

**Figure-3:**
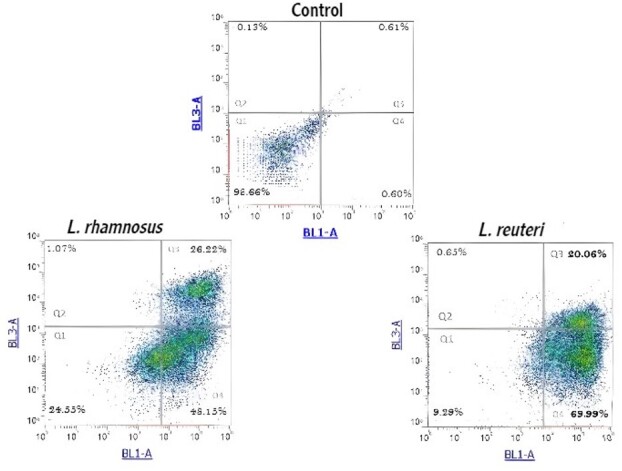
Figure-3. The effects of LR and LGG PMs on Jurkat cells apoptosis

The apoptosis of Jurkat cells treated with IC50 particulate matter PMs from LGG and LR was assessed using flow cytometry (Figure-3). In the group of cells subjected to LGG PMs treatment, a significant increase was detected in the occurrence of both early apoptosis (P=0.007) and late apoptosis (P=0.005) when compared to the control group. Similarly, significant increases were noted in both early apoptosis (P<0.001) and late apoptosis (P=0.033) within the group subjected to LR PMs treatment in comparison to the control group.

## Discussion

The primary objective of the present study was to examine the potential anticancer properties of PMs obtained from LGG and LR on Jurkat cells. The results of our study indicate that the PMs derived from LGG and LR exhibited a time-dependent decrease in Jurkat cell viability. Importantly, these PMs showed limited cytotoxicity towards normal peripheral blood PBMCs. Furthermore, flow cytometry analysis demonstrated a significant increase in both early and late apoptosis within Jurkat cells subjected to PMs. This observation suggests the potential of these mediators in inducing cell death in leukemia cells.

The observed reduction in Jurkat cell viability after treatment with LGG and LR PMs is in line with previous research reporting the anticancer potential of probiotics and their derived products, specifically in leukemia [21][22][23]. Our results are consistent with other studies reporting the anticancer effects of probiotic bacteria and their metabolites on various cancer cell lines, including colon, breast, and esophageal cancer [22][24][25]. It should also be noted that the cytotoxic impacts of Lactobacillus strains on cancerous cells are not limited to their live forms. Studies have shown that heat-killed cells and cell-free L. plantarum and LGG supernatants can reduce cancerous cells’ growth rate [24]. Interestingly, the current study demonstrated that LGG and LR PMs had minimal cytotoxic effects on normal PBMCs, indicating the potential for selective cytotoxicity toward cancer cells without harming healthy cells, consistent with other studies [19][23]. Although some studies have shown that probiotic strains also reduce the growth rate of normal cells [26], the selective cytotoxic effect on cancer cells is crucial for developing targeted therapies that minimize damage to healthy cells, a major challenge in cancer treatment [27]. Flow cytometry analysis demonstrated a significant increase in early and late apoptosis in both groups treated with LGG and LR, compared to the control group. Our study’s results align with other research demonstrating bacterial strains’ apoptotic effects on cancer cells. For instance, Lactobacillus brevis (LB) induced time-dependent apoptosis in Jurkat cells s but not normal human peripheral blood lymphocytes. LB more efficiently induces apoptosis in Jurkat cells than Streptococcus thermophilus [28].

Another study showed that cell-free supernatants postbiotics derived from Saccharomyces cerevisiae var. boulardii have potential antigenotoxic and cytotoxic effects on HT-29 human colon cancerous cells [29]. Despite observing significant effects on cell viability and apoptosis, our study did not find any significant changes in the expression levels of pro-apoptotic (BAX and FAS) and anti-apoptotic (BCL-2 and BCLX) and cell cycle regulator (p27) genes in Jurkat cells treated with LGG and LR PMs. In contrast to our study, when cells were treated with LR, the increased expression of BAX and casp3 genes in HT29 cells and a decrease in Wnt signaling pathway gene expression has been observed, which impacts cell proliferation and differentiation in Kyse30 cells [24][25]. Furthermore, a study on the cytotoxicity of probiotic Lactobacillus spp. found that the probiotic supernatants were cytotoxic to HT-29 and HCT-116 colon cancer cell lines and significantly upregulated cfos and cjun transcripts in these cells [30]. These findings suggest that these PMs’ apoptosis-inducing effects might be mediated through a different molecular mechanism or may be related to post-transcriptional modifications [31]. Further investigation is required to clarify the molecular mechanisms by which LGG and LR PMs exert their anticancer properties.

Our study demonstrated the potential anticancer impacts of PMs derived from LGG and LR on ALL cells, but some limitations should be considered. The study focused on one cancer cell line and one normal cell type, and future research should investigate additional cancer cell lines and normal cell types. Moreover, in vitro experiments may not fully represent the complexities of the in vivo tumor microenvironment, making animal models necessary for further investigation. The specific active components in LGG and LR PMs were not identified, and gene expression analysis was limited to a single time point. Despite these limitations, our study provides preliminary evidence for the potential use of LGG and LR PMs in leukemia treatment. It warrants further research to explore their broader applicability in cancer therapy.

## Conclusion

Findings demonstrate the potential anticancer activity of PMs derived from LGG and LR on ALL cells. The observed selective cytotoxicity towards leukemia cells and induction of apoptosis indicates that these PMs may hold promise as novel natural therapeutic agents in leukemia treatment.

Future research should prioritize investigating the molecular mechanisms contributing to the anticancer effects of PMs and exploring their potential in combination with conventional chemotherapeutic agents to enhance therapeutic outcomes. Additionally, the potential of LGG and LR PMs in other cancer types should be investigated to determine their broader applicability in cancer therapy. It would also be valuable to assess these PMs’ long-term safety and efficacy in preclinical and clinical settings. Identifying the specific active components responsible for their anticancer effects may lead to the development of targeted therapies for leukemia and other cancers, ultimately enhancing outcomes and patients’ quality of life.

## Acknowledgments

This study was supported by grants from the National Institute of Medical Sciences Research, Tehran, Iran (4001382). The funding bodies of the study did not play any role in its design, collection, analysis, data interpretation, and manuscript writing.

## Conflict of Interest

The authors declare no conflict of interest.
